# Retrograde nailing for distal femur fractures in the elderly

**DOI:** 10.1051/sicotj/2015034

**Published:** 2015-12-01

**Authors:** Jasdeep Giddie, Seif Sawalha, Martyn Parker

**Affiliations:** 1 Department of Orthopaedics, Peterborough and Stamford Hospital NHS Foundation Trust, Peterborough City Hospital, Bretton Gate PE3 9GZ Peterborough UK

**Keywords:** Distal femur fracture, Retrograde nail, Union, Complications, Mortality

## Abstract

*Introduction*: We report the results of treating a series of 56 fractures in 54 elderly patients with a distal femur fracture with a retrograde femoral nail.

*Methods*: Fifty-four of the nails were inserted percutaneously with a closed reduction. After surgery all patients were allowed to weight bear as tolerated. Four fractures were supported in a temporary external splint.

*Results*: The mean age of patients was 80.6 years (range 51–103 years), 52/54 (96%) were females. There were no cases of nail related complications and no re-operations were required. One patient was lost to follow up. The 30-day mortality was 5/54 (9.3%) and the one year mortality was 17/54 (31.5%).

*Conclusions*: Distal femoral nail fixation provides a good method of fixation allowing immediate mobilisation for this group of patients.

## Introduction

Distal femur fractures are relatively rare injuries accounting for approximately 1% of fractures in the elderly [1]. They have bimodal age distribution; young patients as a result of high-energy injuries and elderly patients after simple falls [2]. Despite affecting the same anatomical location as young patients, fractures in the elderly pose different challenges due to osteoporotic bone and the overall patients’ medical condition [3]. The aim of surgical management in these frail patients should be considered similar to those with a proximal femur fracture, that is a quick surgical procedure that allows early weight bearing and mobilisation to avoid the complications of prolonged bed rest.

Retrograde nailing is an established management option for these fractures. Previous studies on the outcome of patients managed by retrograde nailing often included both young patients with high-energy fractures and elderly patients with osteoporotic fractures [4–6]. However, the differences in patient and fracture characteristics between these two groups make them two distinct injuries requiring a separate analysis.

We aimed to establish, that the principles of management of osteoporotic distal femoral fractures should mimic the treatment of proximal femoral fractures, i.e. minimally invasive, early surgery with a stable construct to allow unrestricted weight bearing.

We present one of the largest case series of osteoporotic distal femoral fractures managed by the senior author (MJP), a consultant, with a specialist interest in fragility fractures.

## Patients and methods

The inclusion criteria were all patients admitted to Peterborough City Hospital between 1999 and 2014 with a distal femoral fracture treated with a retrograde femoral nail. Any fractures treated with an alternative fixation device and fractures managed non-operatively were excluded from the study. Data was collected using a standard proforma started at the time of the patients’ admission and included documentation of residential status, ASA grade [7] and mobility score. Fractures were classified using the AO system [8]. The main outcomes studied were mortality and the occurrence of fracture healing complications. After discharge from hospital patients were followed up in a specific fracture clinic. One-year mortality rates were determined using local patient register data for the patients.

There were a total of 56 fractures in 54 patients during the study period. Two patients presented with bilateral distal femur fractures. Associated injures were present in two patients. These were an ipsilateral shoulder fracture and ipsilateral ankle. Four patients had a knee arthroplasty in place, three a sliding hip screw in place, two a proximal intramedullary nail and one a hip arthroplasty (Table 1).

**Table 1.** Patient demographics.

Patient numbers	54
Number of fractures	56
Mean age (range)	80.6 (51–103 years)
Female (%)	53 (98.2%)
From own home (%)	40 (74.1%)
Residential home (%)	5 (9.3%)
Nursing home (%)	8 (14.8%)
Fall in hospital (%)	2 (3.7%)
Pathological fractures (%)	4 (7.4%)
Mean haemoglobin g/L	11.7
Mean ASA grade	2.7
No walking aids (%)	19 (35.2%)
One/two sticks (%)	9 (16.2%)
Walking frame (%)	18 (33.3%)
Immobile (%)	8 (14.8%)
AO classification	Number of fractures
33 A – 1	39
33 A – 2	13
33 A – 3	4

For the eight immobile patients, the outcome of surgical management differed, with the emphasis being to allow essential nursing care to be performed without discomfort to the patients.

Surgery was undertaken after informed consent was obtained. The patients were placed supine on a radiolucent fracture table. The knee was placed in 20–30 degrees of flexion with a radiolucent supporting pad placed behind the distal femur. We used image guidance and a radio opaque ruler placed on the patient’s thigh over the fracture site to determine the length and diameter of the nail. Where possible the fracture was reduced closed. A 2–3 cm longitudinal incision was made immediately medial to the patellar tendon and access to the knee either medial to the tendon or using a tendon splitting approach. A guide wire was introduced manually through the intercondylar notch, above the insertion of the posterior cruciate ligament along the distal femur and its position checked under image guidance on two planes. A 12.5 mm hollow reamer was inserted over the guide wire. The selected nail was attached to the targeting device and introduced under image guidance. Distal fixation used the targeting device via stab incisions. For nails greater than 300 mm, proximal locking is achieved free hand from an anterior to posterior direction. For the long nails the aim was to place the tip of the nail in trochanteric region.

**Figure 1.**
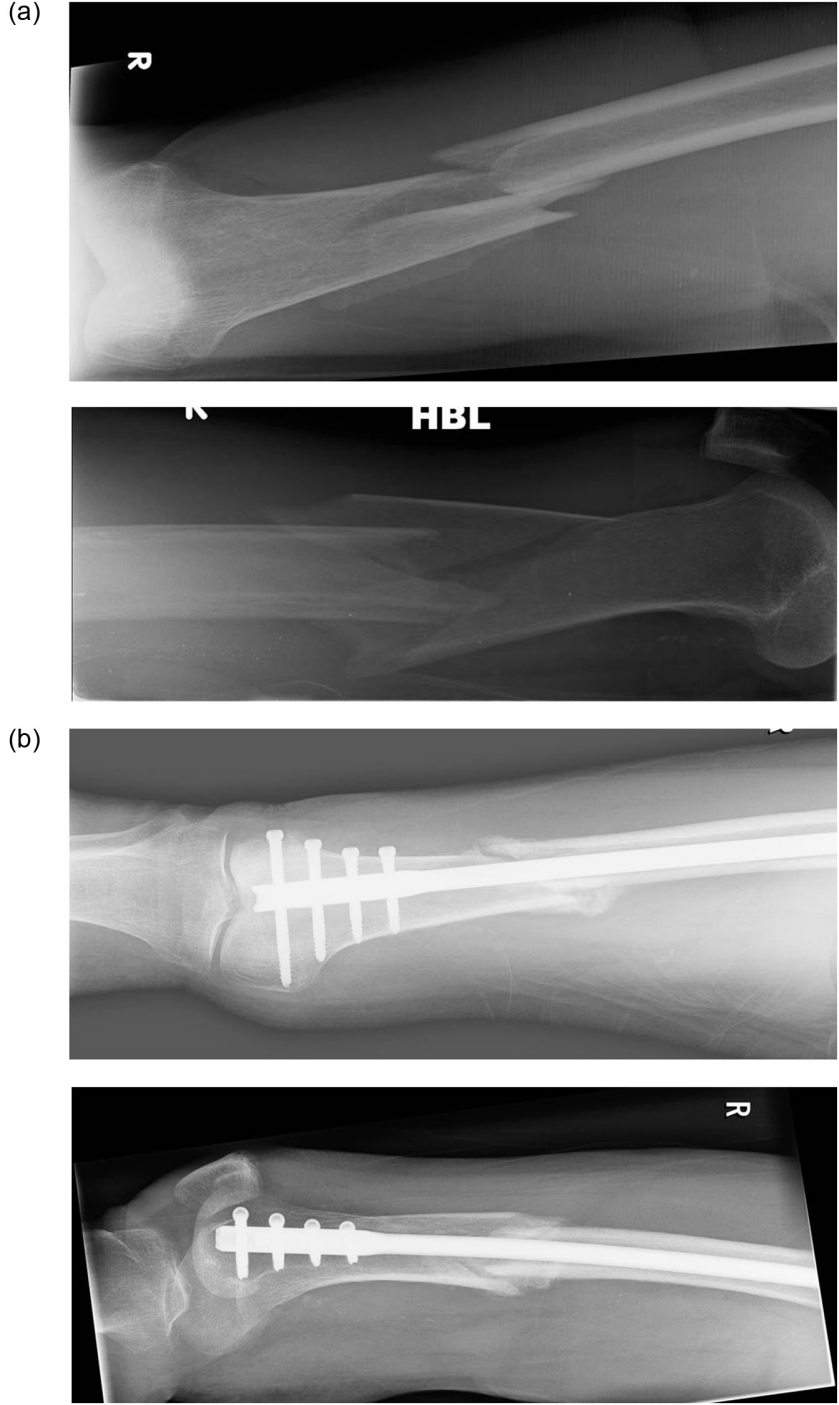
(a) Pre- and (b) post-operative radiographs of distal femur fracture treated with Targon RF nail.

A Targon retrograde nail (RF) for 52 fractures (Figure 1) and a Zimmer retrograde nail for four cases were used. Open reduction of the fracture was required in two fractures, with the remainder being reduced closed. Two distal locking screws were used in two fractures, three distal screws for 11 fractures and four screws for the remainder. Proximal locking with one screw was undertaken for nine fractures and with two screws for 43 fractures. Twelve of the nails were short (length 160–240 mm) and all of them had proximal screws inserted using the alignment jig. The remainder were long nails (range 300 mm–400 mm). For those patients with a knee prosthesis in place the nail was passed between the condyles. For those with a sliding hip screw in place the nail was passed past the distal part of the plate and any screws that impeded passage of the nail were removed percutaneously. The two patients with a proximal femoral nail had this nail removed at the start of surgery.

Surgery was undertaken or directly supervised by the senior author (MJP) for 52 patients. Post-operative management of all the patients was undertaken by the senior author (MJP). For four patients an external splint was used to support the fracture for a period of six weeks. All these fractures were close to the knee joint with limited support from the distal locking screws. All patients were encouraged to mobilise as able with aids and weight bear as tolerated. After discharge from hospital patients were followed up in a specific fracture clinic.

## Results

The mean number of hours to surgery was 38 (range 7–137 h). Mean operative time was 82 min and mean operative blood loss 205 mL. After surgery 17 patients required a blood transfusion. All patients were allowed to weight bear as able. For four patients an external splint was used to support the fracture for a period of six weeks. All these fractures were close to the knee joint with limited support from the distal locking screws. The median hospital stay was 14 days (mean 22.7 days, range 3–76). This included the time spent on any additional rehabilitation wards till the patient was discharged home.

All superficial wound infections were treated successfully with a course of antibiotics (Table 2).

**Table 2.** Post-operative complications.

Superficial wound infection	4
Pneumonia	3
Fat embolism	2
Deep vein thrombosis	1
Post-operative delirium	1

Nail-related complications included one prominent distal screw. This was removed under local anaesthetic as a day case procedure.

Post-operative follow-up was conducted until clinical and radiological union occurred. Mean clinical and radiographic follow-up was 123 days (range 38–365 days). For one patient the fracture line was still visible at one year but no treatment was required. All the other fractures healed uneventfully and no other patient required an additional surgery for the fracture.

**Figure 2.**
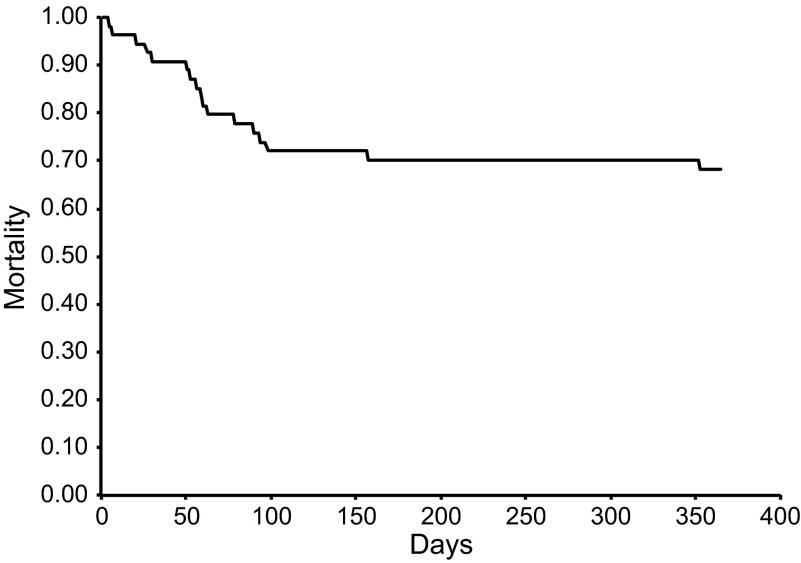
Kaplan Meier one-year survival curve for distal femoral fractures.

Mortality for up to one year from injury is shown in Figure 2. One patient was lost to follow-up at 63 days from injury.

## Discussion

This is a series of elderly patients with distal femur fractures treated with a retrograde femoral nail. Other than the cases of sepsis there were no implant-related complications. This is despite the policy of early mobilisation and the allowing of weight bearing as able. In addition, the surgery was as minimally invasive as possible. This means this type of operative procedure is suitable for frail elderly. We have applied the same principles to this group of patients as we use for the elderly hip fracture patients, that is early technically correct surgery minimally invasive surgery followed by immediate and unrestricted mobilisation.

We did not encounter any implant-related complications in our study. Other researchers reported some complications including femoral fractures above the nail, fracture of the implant, nail protrusion through the knee and fixation failure requiring revision surgery [9–12]. Overall, the mortality rate in distal femur fracture patients was higher in our study than those previous reports [13]. The mean age of patients in our study was higher than those of other studies, which could explain the higher mortality rate.

In this series of elderly patients with osteoporotic distal femoral fractures, we included a 51-year-old male patient. Although this is not considered elderly, the patient was in a nursing home, immobile and sustained a low-energy injury. Given the circumstances through which the fracture occurred he has been included in this study.

The union rate in our study is comparable to published results using other intramedullary devices in elderly patients with distal femur fractures after low-energy trauma. Moskal and O’Shea reported two non-unions in their case series of 14 fractures and Kumar et al. reported one non-union in their case series of 18 distal femur fractures [9, 10]. El-Kawy et al. reported no non-unions in their study of 23 fractures [14]. A significant proportion of our patients died before union could be assessed which reflects the fragility of this population of patients.

There are several limitations to this study. We did not have data on the range of knee movement or functional outcome measures such as post-operative mobility scores. However, this is a frail group of patients who often have a degree of cognitive impairment limiting availability of such data. This is also reflected in the relatively high proportion of patients who died before fractures were followed up to fracture union. A further limitation of this study is that two different types of retrograde nails were used although the design of these nails was very similar.

This paper has three key messages: In elderly patients with an extra-articular distal femoral fracture, surgical treatment using a retrograde femoral nail is reliable. A minimally invasive approach with unrestricted mobilisation has few nail-related complications. Thirdly, despite early intervention there remains a high rate of mortality.

As the numbers of distal femoral fractures continue to increase, future research in this field should continue to develop to treat more complex fracture patterns, using a minimally invasive approach, permit early weight bearing and aim to reduce the significantly high mortality rate.

In conclusion, we have shown satisfactory results using a retrograde femoral nail in the management of elderly patients with a distal femoral fracture. Early and unrestricted mobilisation is possible with a very low risk of fracture healing complications.

## Conflict of interest

JG, SS and MP declare no conflict of interest in relation with this paper.
